# A teaching tool for nursing procedure with oxygen therapy

**DOI:** 10.1080/07853890.2021.1895964

**Published:** 2021-09-28

**Authors:** Maria Rodriguez, Cátia Balsinha, Nataliya Garcia, Valéria Vicente, Ana Cristina Neves

**Affiliations:** a Escola Superior de Saúde Egas Moniz (ESSEM), Egas Moniz Cooperativa de Ensino Superior, Caparica, Portugal; b Centro de Investigação Interdisciplinar Egas Moniz (CiiEM), Egas Moniz Cooperativa de Ensino Superior, Caparica, Portugal

## Abstract

**Introduction:**

Oxygen therapy is applied in a variety of clinical situations where patients have low levels of oxygen in the blood and is part of one of the procedures in nursing care. Knowing that home care is more beneficial for the patient, because it promotes a faster recovery and guarantees comfort and safety, preserving its autonomy [1], it is pertinent to train (enable) both patients and informal caregivers (e.g. family, friends), with the correct knowledge (empowerment) and procedures to follow. Nurses have a fundamental role in the training of patients and/or informal caregivers, knowing their difficulties and realities, thus enabling caregivers to be autonomous in-home care [2–4]. This work presents an original educational tool that uses pictograms to teach and empower patients and/or caregivers to use oxygen therapy materials correctly at home.

**Materials and methods:**

An Educational Tool for Health purposes was originally designed using pictograms, which illustrates/anticipates the therapeutic procedures, destined for patients who need oxygen therapy with regularity. The instrument is made up of a *set of pictograms* that complement the clinical nursing procedure on oxygen therapy (teaching step by step the therapeutic) and a *good practice manual* (helps to understand oxygen therapy and serves as a record and monitoring of the difficulties experienced).

**Results:**

The use of pedagogic tools for health using pictograms can be a strategy to improve interpersonal communication between nurse and the patient and/or informal caregiver [5]. They can also be used as complementary learning tools, because of their universal character, the pictograms constitute an effective way to overcome individual constraints, such as literacy level, familiarity with the images, interpretation, and perception of what is being pictorially represented [6]. The suggested good practice manual is interactive and dynamic, enabling both patient and informal caregiver to focus and actively participate.

**Discussion and conclusions:**

The Strategies for Health Education are increasingly relevant on what concerns the change in health behaviour. Pedagogical materials of this nature facilitate the understanding and use of therapeutic procedures used daily, allowing to reduce the excessive dependence of professionals and health services and are fundamental contributions for a greater autonomy and adherence to the health behaviours of the patient and/or informal caregiver.
